# Bridging event-related potentials with behavioral studies in motor learning

**DOI:** 10.3389/fnint.2023.1161918

**Published:** 2023-04-24

**Authors:** Xueqian Deng, Chen Yang, Jingyue Xu, Mengzhan Liufu, Zina Li, Juan Chen

**Affiliations:** ^1^Guangdong Key Laboratory of Mental Health and Cognitive Science, South China Normal University, Guangzhou, China; ^2^Center for Studies of Psychological Application, School of Psychology, South China Normal University, Guangzhou, China; ^3^Key Laboratory of Brain, Cognition and Education Sciences, Ministry of Education, South China Normal University, Guangzhou, China; ^4^Department of Neurology, Johns Hopkins University, Baltimore, MD, United States; ^5^Department of Cognitive Science, University of California, San Diego, La Jolla, CA, United States; ^6^Institute for Mind and Biology, The University of Chicago, Chicago, IL, United States

**Keywords:** error-related negativity (ERN), feedback-related negativity (FRN), event-related potential (ERP), explicit motor learning, implicit motor learning, task error, sensory prediction error

## Abstract

Behavioral approaches and electrophysiology in understanding human sensorimotor systems have both yielded substantial advancements in past decades. In fact, behavioral neuroscientists have found that motor learning involves the two distinct processes of the implicit and the explicit. Separately, they have also distinguished two kinds of errors that drive motor learning: sensory prediction error and task error. Scientists in electrophysiology, in addition, have discovered two motor-related, event-related potentials (ERPs): error-related negativity (ERN), and feedback-related negativity (FRN). However, there has been a lack of interchange between the two lines of research. This article, therefore, will survey through the literature in both directions, attempting to establish a bridge between these two fruitful lines of research.

## Introduction

Whether it is learning to ride a bike, play a musical instrument, or perform a complex surgical procedure, motor learning is a critical component of our ability to interact with the world around us. It occurs throughout the lifespan and enables us to acquire new skills, refine existing ones, and adapt to changing environmental demands. Through careful experimental designs, behavioral neuroscientists have dissected two parallel processes in motor learning: implicit vs. explicit motor learning. Implicit motor learning refers to the involuntary learning process evoked by movement errors. Explicit motor learning, on the other hand, involves the deliberative adjustment of movement given explicit instructions of the task. Subsequently, a crucial inquiry is what drives each motor learning process. The initial theories have formalized two different kinds of error signals: sensory prediction error vs. task error. Sensory prediction error, the difference between the actual sensory feedback and expected sensory feedback for a given motor command, drives implicit motor learning; task error, the signal that reflects general task performance, drives explicit motor learning. However, recent studies have challenged this view and ask for a more intricate theory for both. A prospective approach is to first nail down the electrophysiological correlates for both implicit vs. explicit motor learning and sensory prediction error vs. task error. At the meanwhile, error-related negativity (ERN) and feedback-related negativity (FRN) are the two heavily investigated motor-related, event-related potentials. Error-related negativity (ERN) is a negative deflection in the EEG signal that occurs in response to the commission of an error during a task. Feedback-related negativity (FRN) is a negative deflection in the EEG signal that occurs in response to the presentation of feedback about the outcome of a task, particularly when the feedback indicates an error. Numerous theoretical frameworks have been proposed to understand these two clearly distinct event-related potentials. However, the behavioral correlates of error-related negativity (ERN) and feedback-related negativity (FRN) still remain controversial and intriguing topics in the field. Hence, in this article, we seek to provide a systematic review of the existing literature on both aspects, and discuss several recent works that endeavor to bridge the two lines of research. By systematically and comprehensively reviewing what we currently know, we aim to create a reference guide that can serve as a valuable resource for future researchers and experimental designs.

## Implicit and explicit motor learning

Experiments reveal that there are two distinct processes when humans learn to counter perturbation: an implicit component, which is an involuntary response to sensory prediction error and which may deteriorate task performance, and an explicit component, which is a deliberative adjustment of aiming direction given explicit instructions from the experimenter. In a canonical experiment of visuomotor rotation, the participant was instructed to reach for a target with a cursor corresponding to the participant’s reaching hand. The cursor path was perturbed by a 45-degree counterclockwise rotation, and the participant was explicitly instructed about the nature of the perturbation. The participant was asked to employ the following explicit strategy: aim for the position that is 45-degree clockwise from the presented target. By applying this strategy, the participant should have been able to eliminate errors. Surprisingly, the performance of the participant was accurate at the beginning of the experiment, but continually deteriorated as the experiment proceeded (Mazzoni and Krakauer, 2006). This result suggests the existence of an implicit learning process distinct from the explicit one of re-aiming.

Jordan and Rumelhart (1992) further analyzed the two processes of motor learning through a novel task design. In their work, the participant was asked to continuously verbally report his aiming direction while learning a visuomotor rotation (Taylor et al., 2014). The explicit learning was measured as the difference between the reported aiming direction and the target. By subtracting this measure from the actual hand position, Jordan and Rumelhart (1992) estimated the magnitude of implicit motor learning. Explicit learning exhibited large fluctuations early in training before settling into smaller adjustments late in training. In contrast, implicit learning was slow and monotonic. This study shows that motor learning is a product of both fast learning of an explicit re-aiming direction and slower implicit learning. It appears that the overall task performance reflects the joint operation of both processes.

The explicit and implicit processes of motor learning were further investigated and observed in many other paradigms, such as mirror reversal where the feedback cursor is mirror reversed rather than rotated (Shadmehr et al., 2010; Krakauer et al., 2019; Kim et al., 2021; Deng et al., 2022). As to implicit motor learning, the cerebellum is commonly regarded as its neural correlate in neurophysiology, while recent studies have indicated involvement of prefrontal, parietal, and premotor cortices as well (Shadmehr et al., 2010; Kim et al., 2021). Explicit strategy is usually regarded as a form of motor planning (Wong et al., 2015; McDougle and Taylor, 2019), and some studies suggest that neural dynamics in motor cortices, primarily PMd and M1, are neural correlates for such motor planning (Kaufman et al., 2014; Wong et al., 2015; Deng et al., 2022). Other studies reveal involvements of premotor and supplementary motor areas (Churchland et al., 2006; Russo et al., 2020). In addition, studies in mice show that motor planning is more global, involving circuits that cover the whole brain; primary circuits include cortico-cortical loops, cortico-thalamocortical loops, and cortico–basal ganglia–thalamocortical loops (Inagaki et al., 2022). The neurophysiology for the two processes still remains unclear; as a result, future application of electrophysiological approaches in complex behavioral experiments have substantive potential to push forward our understanding of implicit and explicit motor learning.

## Sensory prediction error vs. task error

One question that remains is what drives the two processes of motor learning. The two concepts of sensory prediction error and task error have been proposed. Sensory prediction error refers to the difference between the actual sensory feedback and expected sensory feedback for a given motor command (Tseng et al., 2007; Shadmehr et al., 2010; Haith and Krakauer, 2013; Tsay et al., 2022). To bring this to light, visuomotor adaptation occurs when a perturbation is imposed that causes discrepancies in gaze vs. reach directions, leading to a difference between where the arm is seen and where the brain expects to see it based on the motor command. On the contrary, task error refers to the failure to achieve an internally determined task goal and is the signal that reflects general task performance (Taylor and Ivry, 2011; Kim et al., 2019; Leow et al., 2020; Tsay et al., 2022).

It was originally thought that implicit motor learning is driven by sensory prediction errors and that explicit motor learning is driven by task errors (Taylor et al., 2014). To examine the effects of task errors and sensory prediction errors on implicit motor learning, Leow et al. (2020) designed a method, the target jump, in which they could enhance or remove task errors during learning by perturbing target locations; namely, the target location was not in a fixed position. The experimenter was able to manipulate the task error by moving the target position while the participant was reaching (Leow et al., 2020). Implicit motor learning improved when there were task errors, yet did not improve when task errors were eliminated by the target jump. Hence, Leow et al. (2020) argued that task errors were sufficient and necessary to improve implicit motor learning. Morehead et al. (2017) designed another method to dissect the effects of sensory prediction error and task error in implicit motor learning, known as clamped visual feedback. During the clamp, the trajectory of the feedback cursor was hard coded and therefore spatially independent of hand trajectory. The experimenter was able to eliminate task error by always presenting the cursor hitting the target, regardless of how the participant performed. The participants were informed of the nature of the feedback and asked to ignore it. The remaining effect of learning should have been solely due to sensory prediction error. However, the rate and magnitude of implicit motor learning remained the same even across a large range of clamp offsets (Morehead et al., 2017). This result revealed that sensory prediction errors were also sufficient and necessary to drive implicit motor learning.

These results, in fact, prompted further investigation into the relations between sensory prediction errors and task errors. Instead of using the target jump like Kim et al. (2019) and Leow et al. (2020) invented a method meant to simply vary the size of the target. They showed that task error has a modulating effect on learning based on sensory prediction error (Kim et al., 2019). Melding the three methods of, varying target jump, varying target size, and clamped visual feedback, Tsay et al. (2022) showed that task error alone does not drive implicit motor learning; sensory prediction error does, but it is continuously modulated by task error through the course of implicit motor learning.

All things considered then, the story between implicit vs. explicit motor learning and sensory prediction error vs. task error is a more intricate one. Further research may utilize electrophysiology to investigate neural correlates of sensory prediction error and task error. For instance, a starting question could be to ask if sensory prediction error correlates error-related negativity and if task error correlates feedback-related negativity. We will discuss this specific suggestion with further details in our later section. Even if such is not the case, electrophysiology could offer physiological anchors for behavioral frameworks like implicit vs. explicit motor learning or sensory prediction error vs. task error. Further understanding in electrophysiology could inform a more intricate and directed behavioral experimental design.

## Error-related negativity and feedback-related negativity

Both Falkenstein et al. (1991) and Gehring et al. (1993, 2018) groups have independently found error-related negativity in a sentence verification task as well as a key responding task. Ever since, ERN has been found in many other experiments, including Eriksen Noise-Compatibility Task and Sternberg Memory Search Task (Gehring et al., 1995). In general, ERN refers to the large negative deflection in EEG recording at the time of error commission. By subtracting the correct trial average from the incorrect trial average, the EEG signal yields a negative peak at approximately 80 ms after the motor movement onset in the frontal–central regions of the scalp, normally having the most amplitude over the supplementary motor area (Gehring et al., 1993, 1995; Rugg and Coles, 1996; Holroyd and Coles, 2002). The onset of motor movement was usually marked by the electromyograph signal of the corresponding hand.

Feedback-related negativity was found in a time-estimation task by Miltner et al. (1997). The experiment started with a cue when the participants were asked to produce a time interval of 1 s by pressing the button. After 600 ms, the participants received the feedback of correctness for the time interval they produced. The feedback could be in visual, auditory, or somatosensory form. The EEG waveform yielded a similar pattern of negativity to that of ERN. However, three main differences existed between ERN and FRN: (1) FRN was time-locked to the onset of feedback, while ERN was time-locked to the onset of hand movement; (2) FRN peaked at nearly 200 ms after the feedback was given, while ERN did so 80 ms after movement onset; and (3) while ERN was observed at the general the frontal–central regions of the scalp, the scalp distribution of FRN was dependent on the modality of the feedback (Miltner et al., 1997).

Several explanatory models of ERN and FRN have been proposed. Holroyd and Coles believed that ERN was the emergent phenomenon of human reinforcement learning by the anterior cingulate cortex. What they proposed was a “generic” mechanism; i.e., both the ERN and FRN shared the same underlying mechanism of error-processing instantiated by the anterior cingulate cortex (Holroyd and Coles, 2002). Under this notion, there was no real difference between ERN and FRN; in fact, they were both errors that occurred under certain conditions and would be processed and learned under reinforcement learning principles. While Yeung et al. (2004) also had a “generic” notion and believed ERN and FRN originated from the anterior cingulate cortex, they argued the function was not reinforcement learning, but instead, performance monitoring for conflict during information processing. This theory was proposed based on the evidence that ERN could also take place under correct trials (Vidal et al., 2000; Coles et al., 2001). Another possibility was that perhaps a “generic” mechanism for ERN and FRN does not exist; rather, the two signals resulted from completely different processing streams (Heldmann et al., 2008). Jutta Stahl proposed that ERN and FRN were actually internal and external error indicators. While ERN indicated internal errors from our motor system, FRN indicated external errors from outside turbulence. This theory explained several data well. First, the reason for an ERN in a correct trial was that while the overall result of the task was correct, the motor system could still commit errors and, through corollary discharge, be aware of it (Crapse and Sommer, 2008). Second, the time course difference between ERN and FRN was due to completely different processing streams (Stahl, 2010). That is to say that while ERN occurred around 80 ms after movement onset, FRN did so 200 ms after the feedback. However, these behavioral models were based on rather simple behavioral experiments and were limited in their explanatory power and ability to be applied to understand sensorimotor systems in general. Given ERN and FRN are two well-defined experimental ERPs, a promising research direction is to combine ERN and FRN with behavioral frameworks like implicit vs. explicit motor learning and sensory prediction error vs. task error.

## Bridging together

**FIGURE 1:**
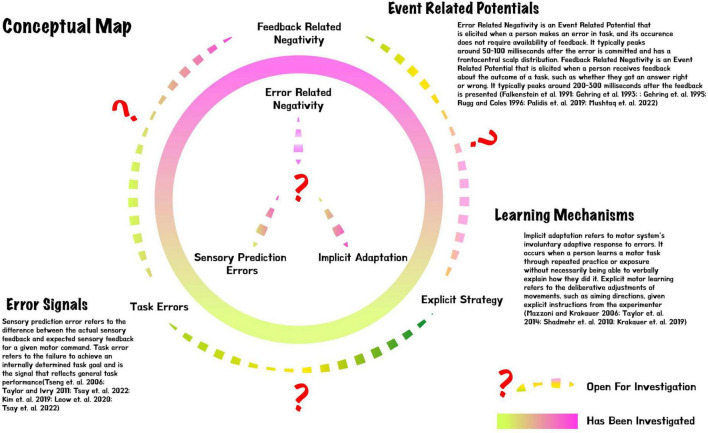
Conceptual map for error-related negativity vs. feedback-related negativity, sensory prediction error vs. task error, and implicit vs. explicit motor learning.

Further, we discuss recent studies that attempt to bridge the behavioral frameworks and electrophysiology aforementioned (Figure 1). A number of electrophysiology studies have been designed under the inspiration of a forward model (Lutz et al., 2013; Joch et al., 2018). A forward model refers to the idea that there exists in our brain a predictor model of future motor consequences based on previously issued motor commands, which is the basis for continuous movement (Jordan and Rumelhart, 1992). It is generally thought that sensory prediction error updates a forward model (Shadmehr et al., 2010). Thus, it is reasonable to suggest that the electrophysiological correlate of updating a forward model is namely the electrophysiological correlate of the sensory prediction error. Lutz et al. (2013) looked into electrophysiology during the development of a forward model for audio-motor associations in playing the piano. Five keys on an electronic piano covered with a specially designed plastic screen were tuned from a diatonic scale into a whole-tone scale. The participant had to select an appropriate key to reproduce the target tone, without knowing, at the beginning, the audio-motor re-mappings. Results showed that the occurrences of ERN decreased along with the development of a forward model for the novel audio-motor mappings, which was behaviorally signified by the decrease in sensory prediction errors (Lutz et al., 2013). It is important to acknowledge that the rationale here is reasoning by correlation. The relationship between ERN and a forward model updating from sensory prediction errors is only suggested by their parallel reduction. Thus, it is more elucidating if the same question could be investigated through recent more cornered behavioral paradigms of forward model and sensory prediction errors, such as the target jump methodology that we have mentioned in the previous section (Leow et al., 2020).

Joch et al. (2018) designed their experiments based on the British pub game, “Skittles,” to ask a similar question: is ERN related to the forward-model predictions of errors? In an experimental trial, the participant would throw the ball with the help of a manipulandum. At 850 ms after ball release, static feedback about the ball flight trajectory together with a verbal cue were presented to provide information about the action outcome. The online feedback of the ball was masked for the experimental group. The results showed that ERN occurred along with a correct prediction of the error from the forward model, before the static feedback was presented and without online feedback, confirming that ERN is an electrophysiological correlate of the forward model (Joch et al., 2018). In short, sensory prediction error is what updates the forward model, and ERN is the electrophysiological correlate of the forward model. However, little effort has been made to explicitly investigate the relationship between sensory prediction error and ERN. Future experimental design that could take these ideas into account. To be specific, researchers who are interested in this issue could consider the following three methods. First, the researchers could use the clamp method to cleanly dissect sensory prediction error in behavioral experiments. In the clamp method, the trajectory of the feedback cursor is hard coded and therefore spatially independent of hand trajectory. Thus, the element of task error could be eliminated and the researchers could cleanly examine the remaining sensory prediction error and its electrophysiological correlates given availability of EEG (Morehead et al., 2017). Another two methods, target jump and varying target size, could also be utilized to eliminate task error and help the researchers to obtain cleanly dissected behavioral substrates of sensory prediction error (Kim et al., 2019; Leow et al., 2020). The operational description of these three methods is given in our previous sensory prediction error vs. task error section.

A recent study should be mentioned here. Matsuhashi et al. (2021) explicitly investigated the roles of ERN in the context of two canonical motor learning experiments. They investigated ERN in a motor sequence learning task and a motor adaptation task (Matsuhashi et al., 2021). They showed that ERN was strongly related with performance improvement in the motor sequence learning task, but not in the motor adaptation task. This is worth noting in that it reveals that even within canonical paradigms of motor learning, empirical findings may differ tremendously across experiments, thereby rendering it difficult for a single theoretical framework to account for all. Nevertheless, several nuances present themselves here. First, Matsuhashi et al. (2021) performed a vertical motor adaptation task. The feedback cursor did not correspond to the veridical reaching hand position. In addition, the vertical screen was a transformed space. The participant must establish a transformation from the participant’s hand position to the cursor position. This transformation was apparently cognitive and explicit. It, therefore, may have been fragile. Non-veridical motor adaptation tasks are indeed different from veridical motor adaptation tasks (Krakauer et al., 2019). Second, motor adaptation was online feedback based. As a result, the error was distributed throughout the participant’s reaching movement. For motor adaptation, there are significant differences between online and endpoint feedback (Shadmehr et al., 2010; Taylor et al., 2014; Brudner et al., 2016; Wang et al., 2022). Consequently, it is worth taking both veridical and online feedback into consideration for future experiment design.

There is also an intricate relationship between FRN and motor learning. van der Helden et al. (2010) conducted a canonical study showing that FRN was predictive of whether subjects learned to avoid an erroneous response the next time the same action had to be performed. In their task, the participants learned a sequence of button presses through trial and error. Each time the participants chose the correct button press, they moved to the next item in the sequence of 12 button presses; on the contrary, if they chose the wrong button, the sequence would restart at item 1 of that sequence. This design allowed van der Helden et al. (2010) to relate FRN amplitude elicited by feedback on a particular button press to performance on that same button press when it was encountered in the sequence. The results showed that the FRN amplitude associated with a mistake was predictive of whether the participants would learn from the mistake, or repeat the mistake (van der Helden et al., 2010). Palidis et al. (2019) designed an experiment that isolated task error and sensory prediction error in motor adaptation, and they used electroencephalography in humans to identify and dissociate the neural correlates of task and sensory prediction error feedback processing. Their experiment was divided into two kinds of conditions. In task error condition, the participants were only presented with binary feedback, successful or unsuccessful, of their reaching toward the target. In the sensory prediction error condition, the sensory feedback, namely the cursor, which would reflect the position of the hand, as well as the target were shown. They observed that the FRN was elicited by binary task error feedback, but not by sensory error feedback (Palidis et al., 2019). This suggests that the process generating the FRN may not be based on sensory prediction error, so a working hypothesis for future experiments is to test whether ERN is the neural correlate for sensory prediction error and whether FRN is the neural correlate for task error. Based on the results of these experiments, further research may be conducted incorporating behavioral frameworks of implicit vs. explicit motor learning. Mushtaq et al. (2022) have conducted a related study on the issue. They designed an experiment that dissected the action process into three stages that would, respectively, elicit reward error, selection error, and execution error. They observed a robust FRN in response to both selection and execution errors, but only the former correlated with behavioral adjustment. In contrast, the amplitude of a positive deflection in the ERP, both before and after the FRN, correlated with choice behavior after execution errors (Mushtaq et al., 2022). This finding suggests a need for a more nuanced interpretation of what FRN represents and how it may be shaped by contextual information.

It is promising that the electrophysiology of sensorimotor systems has been investigated with increasingly complex paradigms from behavioral motor learning and control research. Future research may further take into account sensory prediction error vs. task error as well as implicit vs. explicit motor learning frameworks. Just such a combination may help to solve mysteries on both sides.

## Conclusion

In this article, we have provided a substantive review of the history and literature of both ERN and FRN, the implicit-explicit distinction, and the sensory prediction error vs. task error distinction. We see, of outmost importance, that there should exist a bridge between the two lines of research, and from such will burgeon an ample harvest of scientific discoveries. For instance, one immediate investigation foreshadowed by our discussions is to examine the precise relationship of sensory prediction error and error-related negativity. Research methods could combine previous behavioral experiments for sensory prediction error and electrophysiological experiments for error-related negativity. Subsequent research could be done in a similar manner for task error and feedback-related negativity. Combining insights from these inquiries with our previous knowledge from behavioral experiments of sensory prediction error vs. task error and implicit vs. explicit motor learning, electrophysiological experiments with more sophisticated behavioral designs are open to us for understanding the computational principles and neurophysiological underpinnings of motor learning. Ultimately, we hope that this review will contribute toward a better understanding of the topics, facilitate the development of new conceptual frameworks, and inspire future experimental designs.

## Author contributions

XD drafted the manuscript. CY, JX, ML, ZL, and JC provided critical feedback to it. All authors approved the final version of the manuscript.

## In memoriam

We grieve the passing of our reviewer, Dr. An Wu, on March 16. She was a generous mentor, a young and exceptional scientist, and a rising star in the field of neuroscience.

Dr. Wu’s works have shown us what a beautiful piece of science ought to be: a combination of outstanding methodological innovations, empirical delicacy through clever experimental design, and rigorous reasoning coupled with visionary overarching thesis. Her works not only contribute substantially to our understanding of how our senses of smell and taste are encoded in the brain, but also more generally, give us important insights of how we perceive and how we learn to perceive our world.

Here we highlight some of her remarkable scientific works.

Her work in 2015 introduces large-scale *in vivo* Ca2+ imaging of gustatory sensory afferent neurons to investigate how taste is coded in the brain. The results implicate the dominant coding mechanism of taste: a small number of neurons are “specialists” tuned to single taste quality, while many other neurons are “generalists” who have tuning profiles for two or more taste qualities. These findings fundamentally challenge the “labeled-line coding” hypothesis and show strong support for the “population pattern coding” of taste (Wu et al., 2015). The work is regarded as revolutionary and many researchers have followed up on this inspiring work.

She has also been a force of progress in the field of olfactory sensory coding. Her 2020 work excites us with a method of imaging adult-born granule cells (abGCs) in the olfactory bulb. The results show that the plasticity of young abGCs might be a source of learning for olfactory discrimination. Using optogenetics, she found that feedback from the piriform cortex likely guides plasticity and learning in abGCs. Her findings imply a unique learning mechanism on a neuronal level that relies on adult neurogenesis (Wu et al., 2020a). She proposed a theory suggesting that the plasticity in the olfactory bulb allows for optimal adaptive representation of odors in the dynamic world (Wu et al., 2020b).

We mourn the loss of a brilliant mind. We extend our most sincere condolences to her families, friends, and colleagues. Dr. An Wu will be deeply missed, and her personal and professional legacy will live on.
